# Multi-layered molecular mechanisms of polypeptide holding, unfolding and disaggregation by HSP70/HSP110 chaperones

**DOI:** 10.3389/fmolb.2015.00029

**Published:** 2015-06-05

**Authors:** Andrija Finka, Sandeep K. Sharma, Pierre Goloubinoff

**Affiliations:** ^1^Department of Plant Molecular Biology, Faculty of Biology and Medicine, University of LausanneLausanne, Switzerland; ^2^Laboratoire de Biophysique Statistique, School of Basic Sciences, École Polytechnique Fédérale de LausanneLausanne, Switzerland; ^3^Department of Chemistry, Umeå UniversityUmeå, Sweden

**Keywords:** entropic pulling, HSP40 heat-shock proteins, chaperone proteins, misfolded proteins, conformational diseases, DnaK, DNAJ homologs

## Abstract

Members of the HSP70/HSP110 family (HSP70s) form a central hub of the chaperone network controlling all aspects of proteostasis in bacteria and the ATP-containing compartments of eukaryotic cells. The heat-inducible form HSP70 (HSPA1A) and its major cognates, cytosolic HSC70 (HSPA8), *endoplasmic reticulum* BIP (HSPA5), mitochondrial mHSP70 (HSPA9) and related HSP110s (HSPHs), contribute about 3% of the total protein mass of human cells. The HSP70s carry out a plethora of housekeeping cellular functions, such as assisting proper *de novo* folding, assembly and disassembly of protein complexes, pulling polypeptides out of the ribosome and across membrane pores, activating and inactivating signaling proteins and controlling their degradation. The HSP70s can induce structural changes in alternatively folded protein conformers, such as clathrin cages, hormone receptors and transcription factors, thereby regulating vesicular trafficking, hormone signaling and cell differentiation in development and cancer. To carry so diverse cellular housekeeping and stress-related functions, the HSP70s act as ATP-fuelled unfolding nanomachines capable of switching polypeptides between different folded states. During stress, the HSP70s can bind (hold) and prevent the aggregation of misfolding proteins and thereafter act alone or in collaboration with other unfolding chaperones to solubilize protein aggregates. Here, we discuss the common ATP-dependent mechanisms of holding, unfolding-by-clamping and unfolding-by-entropic pulling, by which the HSP70s can apparently convert various alternatively folded and misfolded polypeptides into differently active conformers. Understanding how HSP70s can prevent the formation of cytotoxic protein aggregates, pull, unfold, and solubilize them into harmless species is central to the design of therapies against protein conformational diseases.

## HSP70/HSP110 evolution and cellular functions

Together with the HSP90s, members of the conserved HSP70/HSP110 family (HSP70s) are prevailing ATP-hydrolyzing chaperones that control all aspects of cellular proteostasis. The HSP70s and co-chaperones may constitute up to 3% of the total protein mass of unstressed human cells (Finka and Goloubinoff, 2013; Finka et al., 2015). In the test tube, proper mixtures of purified HSP70s and co-chaperones can prevent the aggregation of artificially unfolded proteins. This is, however, a rather ineffective process in the absence of ATP, which necessitates a large molar excess of chaperones over their protein substrates (Sharma et al., 2011). In stressed cells, owing to the presence of ATP and J-domain co-chaperones, the HSP70s are much more effective “holding” chaperones. Under regular physiological conditions, they may further use ATP-hydrolysis to warrant the proper *de novo* folding of nascent polypeptides, drive the post-translational translocation of cytosolic polypeptides across membranes and promote their assembly into active oligomers, or drive their disassembly into reversibly inactive conformers (Schuermann et al., 2008; Mattoo et al., 2013). Importantly, HSP70s can forcefully disaggregate stable stress- or mutation-induced misfolded proteins, which may be toxic to animal cells and cause the apoptosis of old neurons in particular, leading to neural tissue degeneration (Bellotti and Chiti, 2008). Moreover, the HSP70s can mediate the refolding of solubilized misfolded polypeptides into harmless native proteins, or control their degradation by chaperone-gated proteases (Mattoo and Goloubinoff, 2014; Cho et al., 2015).

With the exception of exotic thermophilic and hyperthermophilic *archaea*, genes encoding for HSP70s (DnaK in bacteria) are present in all living organisms (Macario and De Macario, 1999). In over 1200 sequenced bacterial genomes, only two members of the order *Aquificales* lack HSP70 genes (Warnecke, 2012). Likewise, all eukaryotic genomes encode for at least half a dozen HSP70 genes and three or four related HSP110 (HSPHs) genes, predicted to be expressed in all the ATP-containing compartments of the cell: cytosol, nucleus, lumen of the *endoplasmic reticulum* (ER), mitochondrial matrix and in plants, the chloroplast stroma and the glyoxisome (Schlicher and Soll, 1996; Wimmer et al., 1997). Moreover, HSP70s can be secreted and exposed on the extra-cellular surface, were they carry a yet unclear ATP-independent immunogenic function, particularly important for cancer detection and therapy (Tytell, 2005).

**Figure 1:**
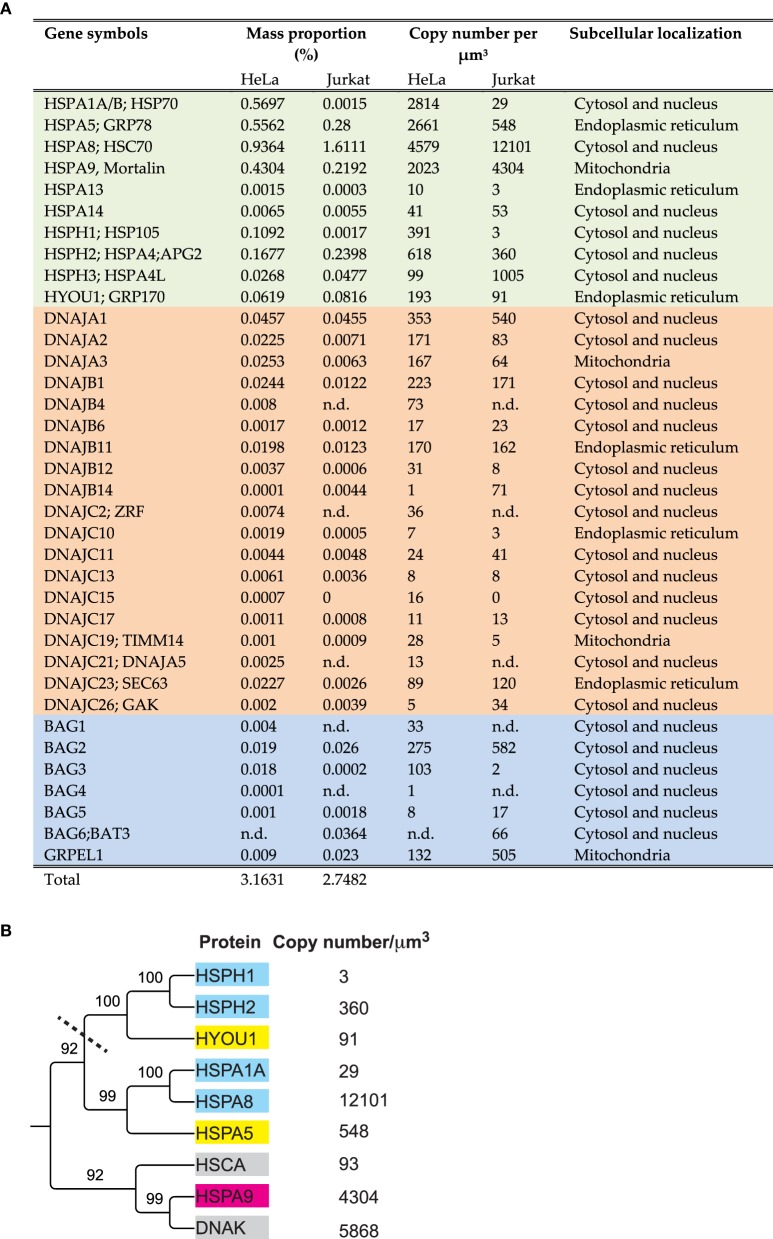
**Amounts, stoichiometries and phylogenetic relationships among HSP70s and their main cochaperones in human cells**. **(A)** List of the significantly detected HSP70 and HSP110 cognate proteins (light green), J-domain cochaperones (orange) and NEFs (blue) in Hela and Jurkat cells and their presumed sub-cellular compartments. **(B)** Phylogenetic tree (neighbor joining method) from the protein sequences of the most abundant HSP70 and HSP110 proteins in Jurkat cells, using *E. coli* DnaK and HSCA as out-groups. Presumed subcellular localization: CYT; cytoplasmic (cyan), ER; Endoplasmic reticulum (yellow). MIT; mitochondria (magenta). The copy number per micron cube are from human (Jurkat) cells (Finka and Goloubinoff, 2013; Finka et al., 2015) and from *E. coli* (gray) (Arike et al., 2012).

A bioinformatic search in the human genome can identify 13 HSP70 and 4 HSP110 genes, which are also actively transcribed. In addition, there are 50 J-domain co-chaperones, known to target the various HSP70s onto their substrates, and at least six nucleotide exchange factors (NEFs) called BAG and GrpE (Kampinga et al., 2009; Finka et al., 2011; Mayer, 2013; Clerico et al., 2015). Some genes are poorly expressed and may remain below detection thresholds of current mass spectrometry methods, or they may be conditionally expressed only in particular tissues, or under various stresses (Finka et al., 2011) such as heat shock (Finka et al., 2015). In Hela cells, high throughput mass spectrometry proteomic using stable isotope labeling of amino acids in cell cultures (SILAC) identified and precisely quantified ten different HSP70/HSP110 proteins, eighteen J-domain cochaperones and five NEFs (Geiger et al., 2012; Finka and Goloubinoff, 2013) (Figure 1A) that summed into 3.2% of the total protein mass. In comparison, a medium resolution mass spectrometry proteomic analysis of human Jurkat cells, identified and quantified ten different HSP70/HSP110 proteins, fifteen J-domain cochaperones and five NEFs (Finka et al., 2015) that summed into 2.7% of the total protein mass.

When exposed to various abiotic or chemical stresses, such as treatments with heat, the HSP90 inhibitor geldanamycin, or various non-steroidal anti-inflammatory drugs, the mRNA and protein levels of cytosolic HSP70 (mainly HSPA1A, HSPA8, and HSPA4) may increase many folds, whereas levels of ER HSP70 (also named HSPA5, BIP) remain unchanged (Saidi et al., 2007; Finka et al., 2011, 2015). Reciprocally, under a ER stress, such as treatments with deoxyglucose or tunicamycin causing a typical Unfolded Protein Response (Ryno et al., 2013), the mRNA levels predominantly of ER BIP may increase many folds, whereas the mRNA levels of cytosolic HSP70 and HSC70 remains unchanged (Finka et al., 2011). The quantitative observation that without stress, HSC70 and BIP are very abundant chaperones, implies that they both carry central house-keeping physiological functions (Finka et al., 2015), beyond their well-characterized defensive role during and following stress (Ben-Zvi et al., 2004; Shorter, 2011; Rampelt et al., 2012; Mattoo et al., 2013).

### HSP70s can assist translation by pulling polypeptides out of ribosomes

At the exit of eukaryotic ribosomes, the L31 protein can bind a special J-domain cochaperone called zuotin (DNAJC2), which in turn can anchor a special HSP70 (SSZ1) and cytosolic HSC70. The presence of several consecutive bulky hydrophobic residues in the sequence of some nascent polypeptides might slow down their translocation through the narrow channel of the ribosome. Remarkably, ATP-hydrolysis by SSZ1 and HSC70 and their consequent clamping onto the emerging polypeptides, alongside their dissociation from their zuotin anchor, can exert a pulling force to relieve the translocation stalling (Otto et al., 2005; Fiaux et al., 2010; Koplin et al., 2010; Shalgi et al., 2013, 2014; Zhang et al., 2014).

### ER HSP70s can pull polypeptides into the ER lumen

Not all ER proteins are synthetized and co-translocated on the SRP-bound ribosomes. Some pre-polypeptides are first synthetized on cytosolic ribosomes. For thermodynamic reasons they may have to reach a near-native fold before being presented to the ER surface to be further translocated across narrow import channels into the ER lumen. On the ER lumen side, BIP (HSPA5) in the ATP-bound state can spontaneously associate to a specific anchoring J-domain cochaperone (SEC63) and thus become poised in the opened state to bind incoming polypeptides from the cytosol. Upon ATP hydrolysis, luminal BIP may “lock” on a threaded polypeptide emerging from the pore and, owing to the concomitant dissociation of ADP-bound BIP from its J-anchor SEC63, the chaperone can exert an entropic pulling force on the locked-upon incoming polypeptide. Pulling of the translocating polypeptide from inside the lumen may compel bulky structured domains that are still on the cytosolic side to collide with the pore entry, leading to their unfolding and unidirectional translocation into the lumen. There, the protein can reach the native state, assisted by soluble DNAJB11 and the ATP-fuelled unfoldases BIP (HSPA5) and HYOU (Matlack et al., 1997; Goloubinoff and De Los Rios, 2007; Griesemer et al., 2014; Melnyk et al., 2015).

### HSP70s can pull polypeptides into the mitochondria

Similarly, in the mitochondrial matrix, a special HSP70 (mortalin, HSPA9) can associate in the ATP-bound state to two anchoring J-domain cochaperones named PAM16/18, and also to a special anchor named TIM44 (Slutsky-Leiderman et al., 2007) and thus await for incoming polypeptides from the cytosol. Given that a steep proton gradient needs to be maintained across the inner mitochondrial membrane, import pores must be as narrow as possible. When a typical hydrophobic polypeptide segment emerges, HSPA9 is triggered to clam upon it, while hydrolyzing ATP and concomitantly dissociating from its membrane anchors. This exerts an entropic pulling force that can unfold the polypeptide on the cytosolic side and unidirectionally translocate it to into the mitochondrial stroma (Goloubinoff and De Los Rios, 2007).

### HSP70s can control protein degradation

HSP70s also control protein degradation by chaperoning various native polypeptides toward specific proteases. Thus, without heat stress, bacterial DnaK, together with DnaJ can specifically bind the native σ32 transcription factor and present it to the protease FtsH for rapid degradation (Arsene et al., 2000). In eukaryotes, HSP70s can direct ubiquitin E3 ligases onto a polypeptide target to be subsequently degraded by the proteasome (Demand et al., 2001; Cyr et al., 2002; Kettern et al., 2010). In addition to chaperone-assisted proteasomal degradation, HSP70s are involved in selective chaperone-assisted autophagy and chaperone-mediated autophagy (Kettern et al., 2010).

### The HSP70s substrates

HSP70s can preferentially bind and process misfolded or alternatively-folded polypeptides, as opposed to (natively-)unfolded and natively-folded polypeptides for which they may have a lower affinity (Priya et al., 2013). By alternatively folded proteins, we intend to emphasize that such substrates can be natively folded and active proteins that also expose specific hydrophobic surfaces with a high affinity for J-domain co-chaperones and HSP70s. Under different biological circumstances, the alternatively folded proteins may acquire differently active, native states without exposed hydrophobic surfaces, thereby having low affinity for the chaperones. Examples of alternatively folded active proteins that can bind chaperones: clathrin cages (Schuermann et al., 2008; Mattoo et al., 2013), IκB oligomers (Weiss et al., 2007), nuclear heat-shock factor-1 trimers (Voellmy and Boellmann, 2007b), nuclear steroid hormone receptors (Echeverria and Picard, 2010), bacterial transcription factor σ32 (Rodriguez et al., 2008); their corresponding, differently active native conformers being, respectively, the clathrin triskelions, IkB monomers, inactive cytosolic heat-shock factor-1, cytosolic steroid hormone receptor and unfolded bacterial transcription factor σ32.

Given the general unfolding activity of HSP70s, it is unclear whether the so called intrinsically unfolded proteins are HSP70s substrates. The intrinsically unfolded α-synuclein protein does not bind DnaK or DnaJ, although it optimally exposes an ideal putative DnaK binding site (Rudiger et al., 1997). In contrast, once α-synuclein is stably aggregated into small oligomers, it strongly and specifically binds DnaJ, which in turn recruits DnaK on the stable misfolded α-synuclein oligomers. Upon ATP-hydrolysis and locking, the oligomers become partially unfolded and disaggregated (Hinault et al., 2010). DnaJ thus guides DnaK specifically onto the misfolded substrate, but not onto the unfolded polypeptide, suggesting that in general, the unfolded conformers might behave as the low-affinity products of the chaperone unfolding reactions.

### HSP70s can control biological switches

HSP70s are key regulators of protein complexes involved in vesicular trafficking. Thus, HSC70 and HSP110, both assisted by the particular J-domain cochaperone auxilin, mediate the ATP-dependent dismantling of clathrin cages for recycling (Augustine et al., 2006; Morgan et al., 2013). Together with the HSP90s, the HSP70s can also control the activation of the steroid hormone receptors (Dittmar et al., 1998) and their own expression under heat stress, by regulating the activity, the oligomeric and phosphorylation state of heat-shock transcription factor-1 (Voellmy and Boellmann, 2007a). It has been reported that the pathological form of the cystic fibrosis transmembrane conductance regulator (CFTR) is stalled with a HSC70/HSP90 complex during protein maturation, suggesting that the HSP70/HSP90 chaperone complexes act transiently more on the early folding steps of the mutant CFTR than in the case of wild type CFTR (Coppinger et al., 2012). HSP70 over-expression in TNF-treated mammalian cells may cause the deoligomerization and thus the inactivation of large active IκB complexes, thereby inhibiting apoptosis (Aschkenasy et al., 2011). In parallel, HSP70s, especially HSPA1A can also inhibit caspases (Sabirzhanov et al., 2012) and are thus attractive drug targets for pro-inflammatory diseases suffering from unchecked apoptosis, such as Parkinson and Alzheimer's diseases. Therapies based on the over-expression of HSP70s, HSPA1A and HSP110 (HSPH1, Apg-1) in particular (Mattoo et al., 2013), might arrest pro-inflammatory degenerative diseases caused by toxic protein aggregates (Hinault et al., 2006). Yet, HSP70s are also pro-oncogenic factors against, which specific inhibitors need to be developed for cancer therapies (Schilling et al., 2015).

HSP70s appear to use a similar mechanism to process both randomly structured aggregates and alternatively folded oligomers: A J-domain cochaperone is first observed to target a HSP70 molecule onto the substrate. This may then trigger ATP-hydrolysis by the HSP70 and its “locking” onto the target polypeptide, thereby causing the local unfolding of the clamped-upon secondary structures and leading to the disaggregation and/or deoligomerization of the substrate. Upon NEF-mediated release, the unfolded substrate may then refold into a differently active polypeptide or be further degraded by an ATP-consuming protease (Goloubinoff and De Los Rios, 2007).

The large diversity of the above-described cellular functions, raises the question of how the HSP70s, which are simple ~640 residue molecules sharing a simple common mechanism, may carry out so diverse cellular tasks? HSP70s are relatively simple two-domain individual 70 kDa polypeptides, which in order to act as effective polypeptide unfolding nanomachines, do not need to assemble into large cage-like oligomers, as in the case of the GroEL/CCT chaperonins, or ring-shaped hexamers in the case of ClpB/HSP104 (Priya et al., 2013; Mattoo and Goloubinoff, 2014). Here, we discuss various non-exclusive molecular mechanisms by which the HSP70s may carry so many diverse housekeeping and stress-damage control tasks in the cells.

### HSP70s nomenclature and phylogeny

As in the case of gene families that have been studied for four decades, the nomenclature of the HSP70s is very complex and confusing with historical layers of terms. For example, the most abundant HSP70 is called DnaK in bacteria, SSA in yeast, HSC70 in humans, whereas they should be called HSPA8 according to the last agreed-upon nomenclature (Kampinga et al., 2009). Similarly, the main ER HSP70, which was traditionally called BIP or Grp78, recently became HSPA5. ER HSP110, which was traditionally called Grp170, is now called HYOU1, although sequence-wise it clearly belongs to the same clade as the cytoplasmic HSPH1, which was traditionally called HSP105 or Apg1 (Figure 1A).

A phylogenetic tree generated from the amino acid sequences of the most abundant HSP70s in Hela cells, HSPA8 (cytosolic HSC70), HSPA1A (cytosolic HSP70), HSPA9 (mitochondrial mortalin) HSPA5 (ER BIP), HSPH1, HSPH2 (cytosolic HSP110s) and HYOU (ER HSP110), shows two large subclasses: The DnaK-like HSP70s, that are both present in bacteria and eukaryotes and the HSP110s (Mattoo et al., 2013), which are only in the cytosol and the ER lumen of eukaryotes (Figure 1B).

### HSP70s structures and functions

**Figure 2:**
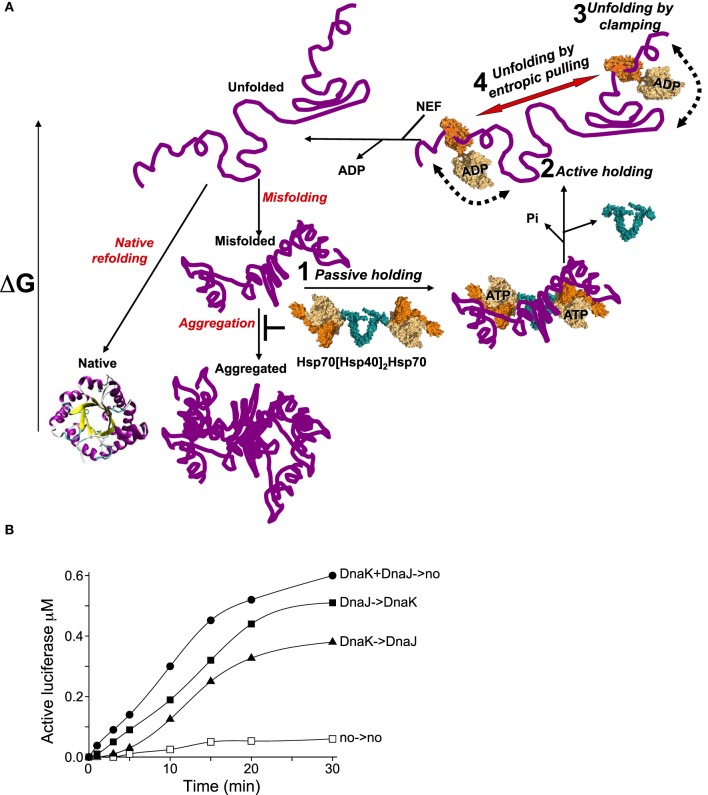
**The various modes of HSP70s action on their polypeptide substrates**. **(A)** A nascent or a stress-unfolded polypeptide (upper left) with high free energy may readily fold to the native state (lower left) or misfold (middle center) and form stable aggregates with a low free energy. HSP70s, possibly pre-associated to HSP40 dimers, can spontaneously bind the misfolding species and thus passively prevent their aggregation, albeit rather inefficiently (step 1). ATP hydrolysis by HSP70 may actively lock the chaperone on the polypeptides and thus effectively prevent their aggregation (step 2). Further, ATP-hydrolysis can cause a forceful clamping motion of the lid toward the base of the PBD and apply an unfolding force that can cause local unfolding of misfolded segments in the substrate (step 3). The concomitant clamping of two distal HSP70s on the same misfolded polypeptide may further cause unfolding by entropic pulling of the intervening misfolded segments between two bound chaperones (step 4). Catalyzed by NEFs, ADP release from HSP70 unclamps the lid from its base. This in turn releases the locally unfolded polypeptide in solution (upper leftward arrow) where is may refold spontaneously to the native state with the lowest free energy. **(B)** Effect of order of addition of DnaK or DnaJ on luciferase refolding. DnaKJ->no (circles): DnaK (0.8 μM) and DnaJ (0.4 μM) were first preincubated for 5 min' with ATP at 22°C and at *T* = 0', FT-Luciferase (1μM) and GrpE (0.7 μM) were added. DnaK-> DnaJ (triangles): DnaK was first preincubated 5 min' with FT-Luciferase and ATP, and at *T* = 0', DnaJ and GrpE were added. DnaJ-> DnaK (plain squares): DnaJ was first preincubated 5 min' with FT-Luciferase and ATP and, at *T* = 0', DnaK and GrpE were added. no->no (empty squares): spontaneous Luciferase refolding without chaperones.

HSP70 and HSP110 are composed of a highly conserved two-lobbed 40-kDa nucleotide-binding domain (NBD) (Figure 2A, light beige) and of a more variable 30–50 kDa protein-binding domain (PBD) (Figure 2A, orange). The highest degree of evolutionary conservation is in the 40 kDa N-terminal NBD, which functions as ATP-fuelled molecular motor regulating dramatic structural changes in the 30 kDa C-terminal PDB (Mayer, 2013). The PDB resembles a crocodile jaw composed of a beta-strand subdomain, acting as a rigid base and a flexible α-helical subdomain acting as flexible lid, across which, bulky misfolded polypeptide substrates can bind and thus become “prays” to be chewed upon. In the apo- and ATP-bound states, the lid is widely open, with its back rear sticking to the NBD, allowing bulky misfolded, or structured alternatively folded polypeptides (such as the transcription factor σ32) with exposed hydrophobic surfaces, to freely bind the hydrophobic surfaces in the wide-open chaperone jaws (Schlecht et al., 2011; Clerico et al., 2015).

## The multilayered molecular mechanisms of the HSP70s

### Mechanism of passive prevention of aggregation

Once a protein is artificially denatured in the presence of a molar excess of HSP70 (and HSP40) but without ATP, it can weakly bind the chaperones and thus become partially prevented from forming large aggregates that scatter light. Hence, many *in vitro* chaperone assays measure the decrease in the net light scattering signal of an aggregating polypeptide in the presence of increasing concentrations of chaperones (Haslbeck and Buchner, 2015). But light scattering intensities are not quantitative and moreover don't reflect the ability of HSP70 chaperone molecules to use the energy of ATP-hydrolysis to change the conformational fate and thus the biological activity of hundreds different polypeptides in the cell.

### Mechanisms of active unfolding by clamping

Upon ATP hydrolysis, the rear of the lid detaches from the NBD and undergoes a closing movement toward the base in a wide rotation motion around the hinge (Figure 2A, step 3). There is experimental evidence that this mere clamping motion may already apply a destabilizing pressure on the misfolded structures of a bound substrate (Sharma et al., 2010). This motion may be futile, either when it is blocked by an over-resistant bulky polypeptide (Schlecht et al., 2011), or when the substrate has escaped the jaws before complete closure has occurred (Sousa, 2012). Importantly, however, this motion may be productive when it reaches complete closure, or “clamping,” allowing the entrapment of a small, fully extended segment of the substrate, typically composed of five-six non-bulky hydrophobic residues preferably flanked by positive charges (Rudiger et al., 2000). Indicative of the complete unfolding of the clamped-upon segment, the ATP-dependent closure of DnaK's PDB has been shown to cause the peptide bonds of the substrate to undergo cis-trans isomerization of their secondary amides, reminiscent of peptidyl prolyl cis/trans isomerase activity (Schiene-Fischer et al., 2002). In the clamped state, the substrate is tightly bound to the ADP-containing HSP70 with an affinity estimated to be about 1000 fold higher than in the ATP bound or apo states, which both display wide-opened PBDs (Goloubinoff and De Los Rios, 2007).

### Mechanisms of active holding

When clamping occurs under heat-shock conditions, substrate dissociation from HSP70s may come to a halt. This is the classic “holdase” activity of the chaperone (Slepenkov and Witt, 2002; Haslbeck and Buchner, 2015). The holding mode of HSP70s may be mediated by the NEFs, in particular bacterial GrpE, which can undergo a reversible thermal denaturation and thus reversibly loose its physiological ability to accelerate ADP- and substrate release from the HSP70s (Diamant and Goloubinoff, 1998; Grimshaw et al., 2001). This “holding” activity of the HSP70s under an ongoing heat-shock makes biological sense, as the untimely release of stress-labile polypeptides would obligatorily lead to their misfolding and aggregation, ultimately into species becoming increasingly resistant to the HSP70s unfolding activity (Sharma et al., 2011) (Figure 2A, step 2). The differences between HSP70-mediated passive and active holding activities were shown in online light scattering measurements during the gradual denaturation and aggregation of a heat-labile luciferase in the presence of prokaryotic DnaK + DnaJ (Sharma et al., 2011), or human HSP70 + HSP40 and HSP110 + HSP40 (Mattoo et al., 2013), in the absence or presence of ATP. Although both chaperone and co-chaperone have specific hydrophobic surfaces that can bind misfolding polypeptides, without ATP, they were poorly effective at preventing the aggregation of the thermolabile luciferase, or in other words, much more of them was needed to prevent luciferase aggregation. In contrast, with ATP, both displayed a strong synergic holding activity at low concentrations, implying that when the conditions are unfavorable, DnaK, Hsp70 or HSP110 can use the energy of ATP hydrolysis to tightly clamp upon the heat-labile polypeptides to actively prevent aggregation and halt their release as long as heat-shock persists. Exactly the same ATP-dependent “holding” behavior was observed with HSP70 (HSPA1A) and HSP40 (Mattoo et al., 2013). The need under a prolonged heat-stress for very large amounts of non-catalytic holding HSP70s to prevent the aggregation of as many polypeptide substrates, could explain why the HSP70s are among the most abundant proteins in human cells (Finka and Goloubinoff, 2013). ATP-fuelled HSP70 clamping may not only cause the local unfolding of the clamped-upon segment but also maintain unfolded also some distal segments that are protruding away from the chaperone jaws.

Remarkably, at physiological temperatures, the clamping by a single DnaK molecule embracing at most 8–10 residues, at any of the dozen or so putative DnaK binding sites (Rudiger et al., 1997) along the 572 residue-long luciferase polypeptide, sufficed to maintain the latter nearly completely unfolded. This was revealed by partial protease treatments: The clamped-upon luciferase species were significantly more sensitive to a limited amount of trypsin, compared to the much more compact resistant misfolded substrates and natively folded products of the chaperone reaction (Sharma et al., 2010). The particular ability of the firefly luciferase to be maintained nearly completely unfolded by a single HSP70 molecule might be attributed to the fact that it folds mostly as a large single domain. In a multi-domain polypeptide, except for the clamped-upon domain that would be maintained unfolded by the chaperone, the other independent domains could be either misfolding or natively folded (Fitter, 2009). Thus, at variance with monomeric misfolded luciferase, aggregation-prone misfolded multi-domain polypeptides, are expected to necessitate the cooperative action of several HSP70 bound at different hydrophobic clusters along the same polypeptide, in order for it to become sufficiently unfolded, to reach after release the native state (Ben-Zvi and Goloubinoff, 2002).

### Mechanism of active catalytic unfolding/refolding

In contrast to heat shock conditions, where the chaperone can be acting as a holding protein platform to prevent aggregation, upon returning to physiological temperatures and GrpE renaturation, the chaperone can switch back into a more effective catalytic mode of action: It can turn into an unfolding enzyme capable to iteratively bind, unfold and release polypeptides, until many substrates become converted into many native products.

Strong evidence that mere ATP-fuelled clamping by a single DnaK molecule may suffice to cause extensive unfolding, leading thereafter to native refolding of the 63 kDa luciferase, was presented by Sharma et al. (2010). Even in the presence of an unprecedented 15 fold molar excess of misfolded substrates over the chaperone, DnaK could effectively regenerate several native luciferase molecules in several consecutive cycles of DnaJ-mediated binding, ATP-fuelled unfolding and GrpE-mediated release (Sharma et al., 2010). At such stoichiometric ratios, chances of concomitant binding of two DnaK molecules to the same polypeptide are excessively low, excluding an obligate cooperative action between them. Given that each stable misfolded luciferase polypeptide that was successfully converted into a native luciferase monomer must have been produced by the action of a single DnaK molecule, ATP-fuelled unfolding by clamping was likely the central mechanism at work in this particular case.

This reaction did set a low consumption record of merely five ATP hydrolyzed per refolded luciferase protein. This strategy, whereby an inactive misfolded polypeptide could be “rehabilitated” by an ATP-consuming unfoldase into a functional one, was estimated to be about a thousand times more economic than the alternative, consisting of degrading it first by an ATP-consuming unfolding protease and then synthetizing a new polypeptide at the cost of three ATPs per peptide bond.

This notwithstanding, the hydrolysis of five ATPs implied that the chaperone may have undergone five productive clamping/unfolding events, but upon HSP70 dissociation, four luciferases spontaneously misfolded again and only one succeeded to reach the native state (Sharma et al., 2010). Yet, there was an alternative scenario, in which the chaperone could have undergone five ATP-hydrolysis events accompanied by five attempted clamping events, but only one would have succeed to reach the native state, whereas the other four would have caused only partial and ineffective unfolding (Sharma et al., 2010). This second scenario was confirmed by an order of addition experiment in which the misfolded luciferase monomers were first interacted with an excess of DnaK, DnaJ and alpha-labeled radioactive ATP for 2 min, to allow initial binding and clamping. Then, apyrase was added to readily convert all the ATP into AMP. Following the separation of the proteins from the nucleotides by a quick spin column chromatography, an inactive chaperone-luciferase complex was isolated that was found to contain 0.6 μM of apyrase-resistant ADP. Remarkably, subsequent addition of GrpE to this inactive complex, readily yield up to 590 nM of native luciferase, although this happened in the total absence of ATP. This experiment produced two important clues about HSP70s mechanism. (1) Whereas the unfolding of a stable misfolded substrate by HSP70s necessitates ATP-hydrolysis, the refolding of the chaperone-released unfolded products does not necessitate ATP and is thus spontaneous. This confirmed Anfinsen's seminal observations (Anfinsen, 1973) that unfolded polypeptides should, in principle, be able the reach their native state unassisted by other proteins. (2) The excellent match between the amounts of entrapped ADP in the isolated inactive chaperone-luciferase complexes, and the subsequently refolded luciferase, implies that upon release, the native refolding was nearly 95% efficient, excluding scenario one, in which only 20% of the released unfolded species would have reached the native state while the other would have misfolded again. Rather, this experiment indicated that four out of five clamping events by the chaperone ended up being unproductive. The possibility that upon ATP-hydrolysis, the ADP-bound HSP70 might undergo only a partial but incomplete clamping and unfolding motion was shown by Mayer and collaborators, who used fluorescence spectroscopy to demonstrate that following ATP hydrolysis, some ADP-bound DnaK molecules need not obligatorily end up being with tightly closed SBDs that clamp around fully extended polypeptides (Schlecht et al., 2011).

Relevant to this chaperone mechanism, quantitative proteomics of human Jurkat cells (Figure 1A) showed copy numbers of 18500:1350:1180, representing an overall stoichiometry of about 16:1:1 for HSP70s, J-domain cochaperones and NEFs (Bags and GrpE), respectively. Assuming an equal distribution of HSP70s through the cytosol of Jurkat cells, this suggests that neither J-domain nor NEF cochaperones can be persistent structural constituents of the core unfoldase machineries. Rather, they may both need to act as substoichiometric catalysts (Pierpaoli et al., 1998; Laufen et al., 1999; Hinault et al., 2010), which in iterative cycles of binding and dissociation, can accelerate respectively, the ATP-fuelled unfolding of the misfolded substrates by the chaperone and the release of the unfolded products from the chaperone (Sharma et al., 2010).

### Mechanism of active unfolding by entropic pulling

In contrast to chaperone-amenable substrates, such as monomeric misfolded luciferase, which could be productively unfolded by the solitary clamping action of individual DnaK molecules, larger soluble aggregates of the same misfolded luciferase (Mattoo et al., 2013), or of heat-denatured G6PDH (Diamant et al., 2000; Ben-Zvi et al., 2004), or of freeze-thawed denatured rhodanese monomers (Natalello et al., 2013), stringently required the presence of a large molar excess of chaperone (DnaK), in order to become productively unfolded and renatured. This suggested that the productive unfolding of severely damaged misfolded polypeptides may require the concomitant binding of several DnaKs at different hydrophobic binding sites along same misfolded polypeptide (Rudiger et al., 1997, 2000). Together with the observed unfolding by pulling effect of mitochondrial HSP70, which is distally applied on translocating polypeptides from within the mitochondria, a common mechanism of unfolding by entropic pulling was proposed that described the forces applied on a misfolded substrate by two or more clamped ADP-DnaK molecules on a misfolded segment situated in between two chaperone-bound segments in an aggregate. Due to the excluded volumes of the two clamped chaperones and their bumping into each other and into the aggregate during a very long time in molecular terms (0.1-1 minute), they were found to apply unfolding-pulling forces, which can pull away and unfold vicinal misfolded segments (De Los Rios et al., 2006; Goloubinoff and De Los Rios, 2007). Thus, in the case of more damaged protein aggregates, the active local clamping of the HSP70s may not suffice to productively unfold a multidomain polypeptide and an additional cooperative mechanism of unfolding by entropic pulling may be necessary (Figure 2A, step 4).

One major limitation of unfolding by entropic pulling is that in order to be effective, more than one HSP70 molecule has to be concomitantly bound to the same misfolded polypeptide. Although as many as 12,000 HSC70 molecules may be present in a micron cube of human cytoplasm (Figure 1) (Finka et al., 2015), some of the remaining 1,200,000 polypeptides may misfold. Once these would exceed 6000, none of them would be reverted to the native state by the 12,000 HSC70s, even at the futile cost of several times 12,000 hydrolyzed ATP.

One possible way for HSP70s to overcome dilution by their own substrate would be to assume that they don't necessarily have to be equally distributed in the cytosol. Moreover they may have a natural tendency to form, at rest, low affinity oligomers (Motohashi et al., 1994). When challenged by misfolded polypeptides, these would readily dissociate and, because of their slow diffusion rate, readily bind nearby each other, increasing the chances of productive multiple binding on the same misfolded polypeptide, despite the unfavorable stoichiometry.

A second way for HSP70s to overcome this limitation would be to function with dimeric J-cochaperone. Indeed all DNAJA/B and HSP40 are dimers associated by a small dimerization domain through their C-terminal ends (Mattoo et al., 2014). Thus, a dimeric DnaJ may optimally present its two N-terminal J-domains for the binding of the NBDs of two ATP-bound HSP70s, while still keeping the two far apart enough so that, after binding to the substrate, they might optimally exclude each other and apply a cooperative unfolding force by entropic pulling. Given that cells have a large excess of ATP over ADP and that the cytosolic HSP70s are about 10 times more abundant than their J-domain cochaperones, it is tempting to speculate that without a stress, most DNAJA/B dimers in the cell will be associated to two ATP-bound HSP70s. Once challenged by a stress-induced misfolded polypeptide, these loose hetero-tetramers could dissociate and the two HSP70s would be thus able to lock onto nearby regions on the same misfolded polypeptide substrate. Simple order of addition kinetics of DnaK-DnaJ-GrpE-mediated refolding of inactive FT-luciferase, illustrates this. When DnaK and DnaJ was first pre-incubated (for 5 min) with ATP and at *T* = 0', supplemented by FTluc and GrpE (Figure 2B, DnaKJ->no), luciferase refolding readily started without a delay and at a rapid rate of 140 nM per 5 min. When DnaJ alone was first preincubated with TFluc and ATP at *T* = 0', DnaK and GrpE were added (Figure 2B, DnaJ->DnaK), refolding was slightly delayed and the refolding rate was slightly lower (90 nM in the first 5 min). In contrast, when DnaK alone was first preincubated with ATP and FTluc, and at *T* = 0' incubated with DnaJ and GrpE (Figure 2B, DnaK->DnaJ), luciferase refolding was strongly delayed and reached less than a net 15 nM active Luciferase in the first 5 min of the reaction. These results indicate that, as initially shown by Bukau and colleagues (Gamer et al., 1992), the DnaJ dimer may indeed be the first to bind the substrate and thereafter effectively recruit two DnaKs onto the substrate. Noticeably, when DnaK was compelled to pre-interact first with the substrate, the strong delay in the refolding rate suggests that some time was needed for the wrongly associated DnaKs to first dissociate from the substrate and for DnaJ to properly bind and only then, optimally recruit DnaK on the substrate. Moreover, the fastest rate that we observed with preincubated DnaK + DnaJ + ATP prior they were presented to the substrate and GrpE, suggest that in the presence of ATP, DnaK and DnaJ may form a loose hetero-oligomer, likely a DnaJ_2_DnaK_2_, optimally poised to bind cooperatively to the same misfolded substrate and engage in the HSP70-unfoldase cycle (Figure 2A, step 1).

A third way to overcome the limitation for unfolding by entropic pulling caused upon dilution of the HSP70s by their own substrate, would be the spontaneous formation of loose hsp70dimers. Thus, HSP110:HSP70 heterodimers associated by their N-terminal domains can apparently form and act reciprocally as NEFs on each other. Somehow, together they can efficiently disaggregate large stable aggregates (Schuermann et al., 2008; Mattoo et al., 2013). Similarly, bacterial DnaKs might also form loose homodimers, which would favor their ability to act upon resistant substrates by entropic pulling (Sarbeng et al., 2015).

Finally, one elegant way in which evolution may have chosen to overcome HSP70 limitation by substrate dilution, was recruitment of powerful disaggregating nanomachines, such as ClpB in *E. coli* or HSP104 in yeast. These are AAA+ type hexameric cylinders capable of applying ATP-fuelled power-stokes on inserted misfolded polypeptide loops from the aggregates in the hexamer cavity and unfold them by stretching strokes (Goloubinoff et al., 1999; Winkler et al., 2012). Once activated by HSP70, ClpB or HSP104 can convert large protein aggregates into lower oligomeric species amenable to HSP70-mediated unfolding by clamping.

The multilayer mechanisms of HSP70 as holding and unfolding chaperones can be summarized in four steps (Figure 2A). Without ATP, the wide opened protein-binding domain may weakly bind to hydrophobic patches on the surface of misfolding polypeptides, thereby passively and rather inefficiently preventing aggregation (Figure 2A, step 1). But passive binding is probably irrelevant to the physiological situation in cells. ATP-hydrolysis, by driving the closure of the PBD in the substrate and causing local unfolding, can dramatically increase the affinity of the misfolded polypeptides for the chaperone, thereby actively and efficiently prevent aggregation (Figure 2A, step 2). Owing to the transient thermal inactivation of the NEF (GrpE), dissociation during heat-shock is prevented. Upon returning to the physiological conditions, GrpE may become functional again and trigger the release of the unfolded substrate, leading to efficient spontaneous refolding. Catalytic cycles of misfolded substrate binding, ATP-fuelled unfolding by clamping, release and spontaneous refolding may thus convert non-aggregated misfolded species (Figure 2A, step 3), such as FT-luciferase into native proteins (Sharma et al., 2010). In case the misfolded species are tightly associated into large insoluble aggregates, the binding and local unfolding by clamping of a single Hsp70 molecule may not suffice to unfold distal misfolded segments in the same polypeptide. The binding and locking of additional HSP70 molecules elsewhere on the same polypeptide would add another unfolding effect by way of the Brownian movements of the locked chaperones and their volume exclusion from each other, applying unfolding forces on the misfolded segments located in between the HSP70-bound sites (Figure 2A, step 4).

This additional mode of ATP-dependent unfolding by distal entropic pulling of the HSP70, can predominate in the case of polypeptide precursors that are partially folded in the cytosol and need to be imported across the narrow pores of the mitochondria (Elsner et al., 2009), the ER (Matlack et al., 1997) and chloroplasts (Shi and Theg, 2011). Here, the organellar ATP-bound HSP70s are uniquely targeted to the import pores by specific pore-associated J-domain cochaperones to clamp upon the entering polypeptides segments, which are unstructured. In these cases, the unfolding action of the organellar HSP70s is not local but distal, by pulling on the bulky structures that are still on the cytoplasmic side of the precursor protein and are forced to collide onto the narrow pore entry and thereby to unfold prior translocation. Moreover, ATP-dependent unfolding by entropic pulling is likely to be the mechanism by which HSC70 and HSP110, targeted by their specific J-domain cochaperone auxilin, actively deoligomerize clathrin cages into triskelions (Zhuo et al., 2010).

## Conclusion

HSP70s are a central hub of the chaperone network carrying very diverse physiological functions in proteostasis, such as polypeptide folding/unfolding, protein assembly/disassembly, protein activation/inactivation, polypeptide translocation, and degradation. Moreover, HSP70s act as polypeptide-unfolding nanomachines that serve as defenses against stress- and mutation-induced formation and accumulation of cytotoxic misfolded protein conformers. Assisted by J-domain cochaperones, the HSP70s can specifically detect among a large excess of low-affinity native proteins a minority of high-affinity misfolding intermediates on their way to form toxic aggregates. HSP70s may further collaborate with each other (DnaK-DnaK, HSP70-HSP110) and with AAA+ unfoldases (HSP100/ClpB), at disentangling already formed aggregates and convert them into natively refoldable, or protease-degradable polypeptides. The pharmacological upregulation, or adenoviral-mediated over-expression of specific HSP70s, HSP110s and their specific J-domain and NEF cochaperones, are attractive avenues for therapies against degenerative protein conformational diseases and aging (Ebrahimi-Fakhari et al., 2013). Moreover, the development of specific inhibitors of HSP70s and J-domain co-chaperones, is a promising approach to develop therapies against aggressive cancers (Patury et al., 2009; Chang et al., 2011).

### Conflict of interest statement

The authors declare that the research was conducted in the absence of any commercial or financial relationships that could be construed as a potential conflict of interest.
